# Supplementary material for: Entropic patchiness drives multi-phase coexistence in discotic colloid–depletant mixtures
Source: Sci Rep. 2017 Dec 6;7:17058. doi: 10.1038/s41598-017-16415-0 (PMC5719020; doi:10.1038/s41598-017-16415-0)
Supplement: Supplementary file 1 — Supplementary information [file 41598_2017_16415_MOESM1_ESM.pdf]

Entropic patchiness drives multi-phase coexistence in anisotropic colloid–depletant mixtures

Supplementary information

Á. González García [1,2] , H.H. Wensink [3] , H.N.W. Lekkerkerker [2] , and R. Tuinier [1,2,*]

[1] Laboratory of Physical Chemistry, Department of Chemical Engineering and Chemistry, & Institute for Complex Molecular Systems (ICMS) Eindhoven University of Technology,P.O. Box 513 , 5600 MB, Eindhoven, The Netherlands

[2] Van 't Hoff Laboratory for Physical and Colloid Chemistry, Department of Chemistry & Debye Institute, Utrecht University, Padualaan 8, 3584 CH, The Netherlands

[3] Laboratoire de Physique des Solides - UMR 8502, Université Paris-Sud and CNRS, 91405 Orsay Cedex, France

[*] r.tuinier@tue.nl

In this supplementary information, we first provide a 3D plot which helps understanding Fig. 2 of the main text. Then, we show a comparison with the hybrid simulation—theory model by Zhang et all [49], which follows the same trends explained in the main text but for a lower depletant—to—disc diameter ratio.

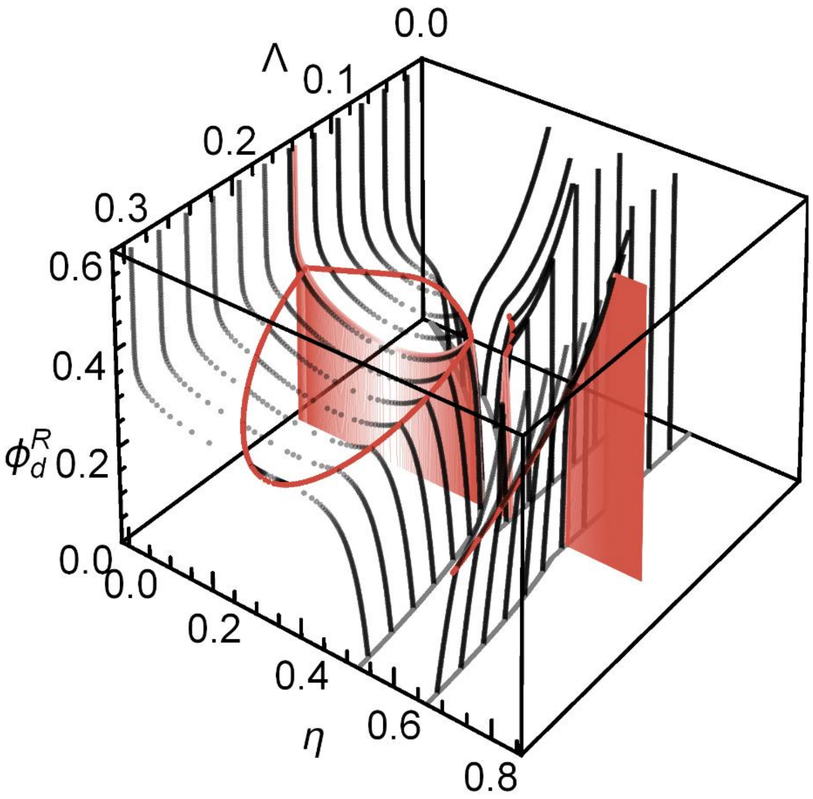

Figure SI 1: Collection of phase diagrams fixing the depletant—to—platelet diameter as q=0.2 using the depletant concentration in the reservoir. In clear gray the phase diagram in the absence of depletants is presented. The triple point lines of the I-I-N and I-I-C are presented as a red curve, also for the platelet aspect ratios not presented in this plot. The red plane corresponds to the phase diagram at which a quadruple point is present for this q. The top view of this plot can be identified with the curves corresponding to q=0.2 in Fig. 2 of the main text.

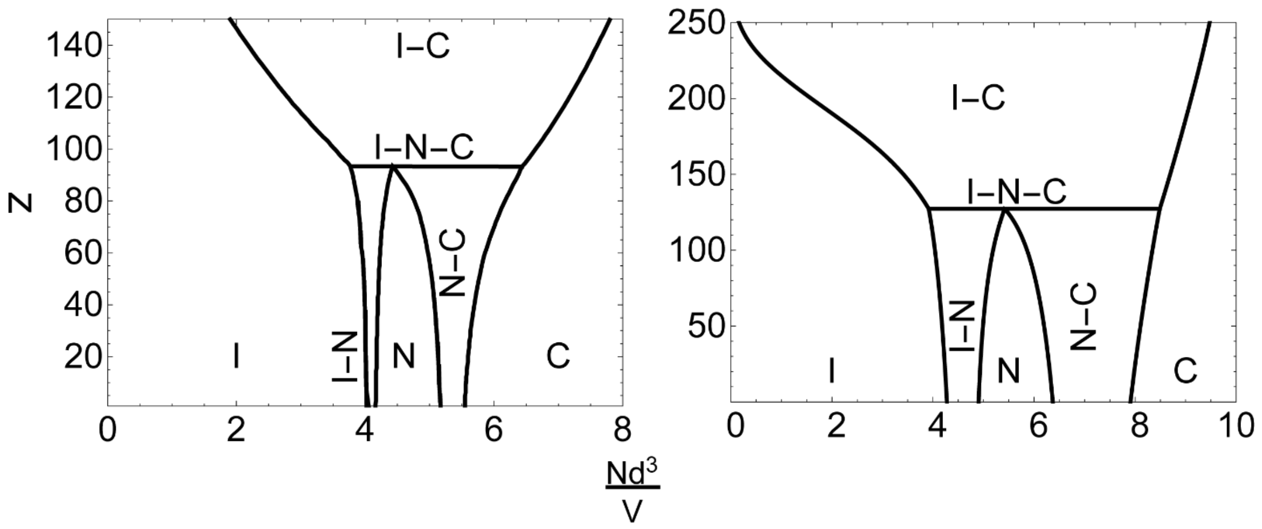

Figure SI 2: Comparison of computed phase diagrams for a mixture of colloidal platelets plus nonadsorbing polymers (simplified as mutually penetrable hard spheres) $\Lambda = 0.1$ and $q = 0.1$ Left: hybrid approach of Monte Carlo computer simulation and theory for cut spheres plus depletants [49]. Right: our free volume calculation results for discs plus depletants.
